# Unsupervised gene selection using biological knowledge : application in sample clustering

**DOI:** 10.1186/s12859-017-1933-0

**Published:** 2017-11-22

**Authors:** Sudipta Acharya, Sriparna Saha, N. Nikhil

**Affiliations:** 1 IIT Patna, Department of Computer Science and engineering, Patna, India; 2 IIT Ropar, Department of Computer Science and engineering, Punjab, India

**Keywords:** Feature selection, Gene Ontology (GO), Sample classification, Gene-GO term annotation matrix, Multi-objective clustering

## Abstract

**Background:**

Classification of biological samples of gene expression data is a basic building block in solving several problems in the field of bioinformatics like cancer and other disease diagnosis and making a proper treatment plan. One big challenge in sample classification is handling large dimensional and redundant gene expression data. To reduce the complexity of handling this high dimensional data, gene/feature selection plays a major role.

**Results:**

The current paper explores the use of biological knowledge acquired from Gene Ontology database in selecting the proper subset of genes which can further participate in clustering of samples. The proposed feature selection technique is unsupervised in nature as it does not utilize any class label information in the process of gene selection. At the end, a multi-objective clustering approach is deployed to cluster the available set of samples in the reduced gene space.

**Conclusions:**

Reported results show that consideration of biological knowledge in gene selection technique not only reduces the feature space dimensionality in great extent but also improves the accuracy of sample classification. The obtained reduced gene space is validated using strong biological significance tests. In order to prove the supremacy of our proposed gene selection based sample clustering technique, a thorough comparative analysis has also been performed with state-of-the-art techniques.

## Background

Analysis of microarray gene expression data plays a key-role in solving several problems related to the field of bioinformatics like cancer or other disease diagnoses, which help to make the plan for appropriate treatment technique for patients. Clustering [1] and bi-clustering [2] of tissue samples are some strong data mining strategies to do such analysis. With the increase in the available biological information, the gene space is also becoming huge. The analysis of gene expression data becomes infeasible and complex in the presence of high dimensional gene space. Thus the immediate solution could be to reduce the gene space by attentively selecting the relevant subset of genes from the large collection of genes. The selected subset of genes can further take part in delicately clustering the available set of samples. The effectiveness of gene selection in the analysis of gene expression data sets is supported by various state-of-the-art research studies [3, 4]. The existing gene selection approaches can be either supervised [5] or unsupervised [6] depending on the use of actual class label information during the gene selection process. Supervised gene selection techniques [5] are widely applied but less attention is given in developing gene selection techniques using unsupervised learning [6].

Grouping semantically related genes using biological knowledge extracted from existing databases is an emerging field of research in recent years. A genuine source of such biological knowledge is Gene Ontology(GO) (http://www.geneontology.org/). To describe cellular functions of proteins and genes, a potential dynamic vocabulary is Gene Ontology(GO). The GO comprises of three ontologies which are, Biological process(BP), Cellular component(CC) and Molecular function(MF). Each of them is a complete ontology containing several processes and sub-processes, which are referred as *GO terms* having direct and indirect relationships with each other. Genes from various organism databases are annotated with specific GO terms and are available for download from the GO website (http://www.geneontology.org/). It is increasingly gaining interests in defining functional relatedness using “semantic similarity” of genes based on GO annotations [7–9]. In several literatures [10–12] authors have proposed different gene-clustering methods based on GO based similarity measures. Though biological information of GO rigorously has been used for grouping semantically related genes, but in the field of gene selection the usage of biological knowledge extracted from GO database has not been explored much.

Motivated by this fact, in this paper we have proposed an unsupervised feature selection technique utilizing biological knowledge extracted from GO. Here as biological knowledge we have used gene annotation data.

### Related works and motivation

There are several existing works on development of feature selection algorithms. For example, Yang et al. proposed the methods for gene selection (GS) namely GS1 and GS2 which can handle unbalanced sample class sizes and no explicit statistical model on the gene expression values was considered by them [13]. Tsai et al. [14] proposed an innovative generalization of signal-to-noise ratio (SNR) for multiclass cancer classification. In [15], Liu et al. proposed a method combining statistical similarity measure and supervised learning named as recursive feature addition (RFA) for feature(gene) selection. A feature selection approach termed as effective range based gene selection (ERGS) is proposed by Chandra and Gupta [16]. Genetic algorithm based feature selection was introduced by Gunavathi and Premalatha [17]. In Saha et al. [18] authors have proposed multi-objective (MO) semisupervised clustering as well as feature-selection technique called SemiFeaClustMOO which encodes feature combination and the set of cluster centers in the form of a string.

All the above mentioned feature selection techniques do not explore biological knowledge for designing the gene selection algorithm. But the use of biological knowledge could be a potential source for designing alternative feature selection methods. For example in [19], authors have proposed a GO based feature selection method where they have developed a hybrid similarity measure between genes using both semantic similarity extracted from GO and Pearson distance. Further they have used feature selection technique, HykGene, and Minimum Redundancy Maximum Relevance (MRMR) with proposed hybrid similarity measure on two data sets.

In [20], authors have proposed a feature selection method utilizing biological knowledge followed by clustering of samples on gene expression data. They have adopted CLARANS (Clustering Large Applications based upon RANdomized Search) for feature(gene) selection. Medoids of different biologically enriched obtained gene clusters are chosen as members of the reduced feature set. A similar work has been done in [21] where instead of CLARANS, a fuzzy clustering technique, FCLARANS, has been adopted for feature selection.

In this paper we have proposed a novel unsupervised gene selection based sample clustering technique utilizing gene annotation information available at GO database. The annotation data for each gene contains the complete information about the processes and the sub-processes for which the gene is responsible. Two genes having same annotation patterns signify that both of them are involved in similar processes and sub-processes. Here genes are represented as features. So throughout this article we have used the word ‘gene’ and ‘feature’ alternatively. The proposed technique first performs unsupervised feature selection to reduce the dimensionality of large gene space of microarray data using annotation information of genes retrieved from GO. Performing feature(gene) selection in the proposed way guarantees to generate a set of most informative, semantically discriminative set of genes. This obtained feature/gene set is biologically validated using existing GO tool. In the second step, a multi-objective clustering technique is applied on samples of microarray data over the reduced gene-set to partition the samples into some homogeneous groups. Finally. different comparative analyses of the obtained results with existing state-of-the-art techniques are carried out to illustrate the power of the proposed gene selection based sample clustering technique.

## Methods

Our proposed unsupervised gene selection based sample clustering technique can be divided into two modules which are as follows,

- In the first module we have proposed an unsupervised feature selection technique utilizing gene annotation data of GO to select most informative and semantically discriminative set of genes. Several biological validation tests are also performed to get most biologically enriched feature(gene) set.
- In the second module we have investigated the utility of proposed feature/gene selection method by performing a multi-objective based clustering on samples of gene expression data over both original and reduced gene space. A rigorous comparative study has been performed for this purpose.

**Fig. 1:**
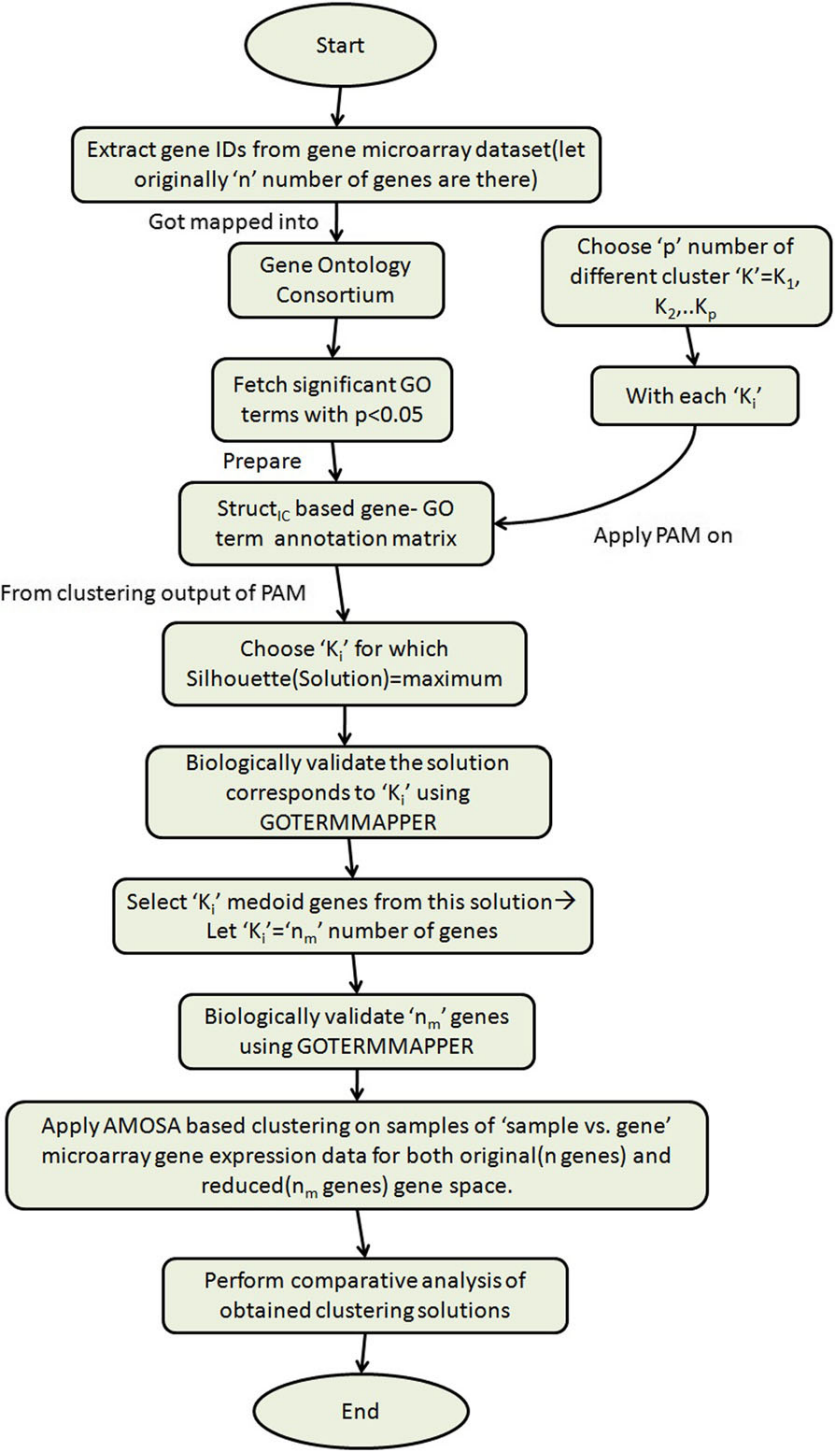
Flowchart of the proposed framework

The flowchart of the proposed gene selection based sample clustering technique is shown in Fig. 1. A detailed description of the overall proposed methodology is given below.

### Module 1: feature selection and partitioning around medoids (PAM)

This is the very first module of the proposed feature selection methodology. At first gene-GO term annotation matrix corresponding to a chosen gene expression data set is formed using knowledge of GO (http://www.geneontology.org/). Next on the prepared annotation matrix, PAM clustering algorithm is applied to get groups of semantically related genes. Note that our proposed feature selection technique is unsupervised in nature so no class label information is used in it. Following tasks are performed in this module.

#### Preparing gene-GO term annotation data for PAM based clustering

As our proposed feature selection method utilize the biological knowledge from GO only, therefore, instead of gene-expression data gene-GO term annotation data is considered in it. For a chosen data set GO tool like Gene Ontology consortium^1^ is used to annotate genes by one or more GO terms. From the annotation data significant GO terms i.e., GO terms having degree of functional enrichment (*p*-value) < 0.5 are chosen for further analysis. Next two tasks as mentioned below are performed,

1. Calculation of structure based information
  content(*Struct*_*IC*_) for all mapped significant GO terms.
2. Creation of gene-GO term annotation matrix using *Struct*_*IC*_ of each GO term.

**1) Calculating structure based information content of mapped GO terms:**

The information content (IC) [22] of a GO term is related to how often the term is applied to genes in the database, such that rarely used terms are ascribed higher IC values. So it can be treated as a measure of importance of GO terms. IC can be of two types, Corpus based IC [23] and Structure based IC [23]. The corpus based IC of a GO term depends on how many number of genes are annotated with that term. But according to [24], IC of a GO term should be independent of the annotation distribution of that term. Because it suffers from corpus bias and semantics of a term can not be measured properly.

Inspired by this fact, authors of [23] have proposed a structure of GO based IC measurement methodology where both level and the number of descendants of a GO term are considered while computing its IC. It is based on the convention that, IC of a term is dependent on it’s depth in GO tree. IC value increases with increase in the depth of a term as it contains more specific information. Also it depends on another factor i.e., the number of descendants of a term. The more number of descendants means less specific information. Depending on these factors authors of [23] have proposed a structure based IC of a GO term. The full GO tree^2^ topology is needed for this calculation. It is calculated as follows,

1 $$ Struct_{IC}(t)=depth(t) \times semantic\_coverage(t)  $$

where, the maximum depth of a term is taken as its depth, and $semantic\_coverage(t)=\left (1-\frac {log(desc(t)+1)}{log(total-terms)}\right)$ is a function of number of descendants of the term. According to this formula, overall semantic coverage of a term having less number of descendants is more.

In the above mentioned way the *Struct*_*IC*_ values for all of our obtained significant GO terms are calculated.

**2. Creating gene-GO term annotation matrix using** ***S*** ***t*** ***r*** ***u*** ***c*** ***t***_***IC***_ **of each GO term:**

Suppose for biological, molecular and cellular components, for an input set of *n* genes, total significant GO term-counts are *x*, *y* and *z* respectively. Thus a matrix of size *n*×(*x*+*y*+*z*) is generated. Entries in the matrix are either ‘0’ or ‘ *Struct*_*IC*_’ value of the corresponding GO term based on the condition that the gene is mapped to that particular GO term or not. Each row of an annotation matrix is a weighted gene-GO term annotation vector. Mathematically it can be described as follows:

If ∃ n genes and x, y, z number of significant Biological function GO terms, Molecular function GO terms and Cellular component GO terms, respectively, then |*M*| = *n*×(*x*+*y*+*z*).

Suppose *G*_*i*_ represents *i* ^*th*^ gene where *i*∈ [1,*n*].

*Bio_GO*_*k*_ represents *k* ^*th*^ significant term of Biological process ontology, where *k*∈ [1,*x*].

*MF_GO*_*l*_ represents *l* ^*th*^ significant term of Molecular function ontology, where *l*∈ [1,*y*].

*CC_GO*_*m*_ represents *m* ^*th*^ significant term of Cellular component ontology, where *m*∈ [1,*z*].

The entries of annotation matrix are computed as follows,

$$ M[i][{Bio\_GO}_{k}]=\left\{ \begin{array}{ll} Struct_{IC}({Bio\_GO}_{k}),& \text{if}\,\, G_{i} \\ & {annotated}\\ & \text{with} \\ & {Bio\_GO}_{k}\\ 0, & \text{otherwise} \end{array}\right. $$

where *i*∈ [ 1,*n*] and *k*∈ [1,*x*].

$$ M[i][{MF\_GO}_{l}]= \left\{ \begin{array}{ll} Struct_{IC}({MF\_GO}_{l}), & \text{if}\,\, G_{i} \\ & {annotated}\\ & \text{with}\\ & {MF\_GO}_{l} \\ 0, & \text{otherwise} \end{array}\right. $$

where *i*∈ [ 1,*n*] and *l*∈ [1,*y*].

$$ M[\!i][{CC\_GO}_{m}]= \left\{ \begin{array}{ll} Struct_{IC}({CC\_GO}_{m}),& \text{if}\,\, G_{i} \\ & \text{annotated}\\ & \text{with} \\ & {CC\_GO}_{m} \\ 0, & \text{otherwise} \end{array}\right. $$

where *i*∈ [ 1,*n*] and *m*∈ [ 1,*z*].

After generation of annotation matrix, the distance between two gene annotation vectors is measured using three well known distances alternatively, viz. Euclidean [25], City block [25, 26] and Cosine distance [25] as demonstrated in the following equations.

2 $$ {}Eucli_{struct}(G_{i},G_{j})=\sqrt{\sum_{p=1}^{x+y+z}(M[i][p]-M[j][p])^{2}}  $$

3 $$ City_{struct}(G_{i},G_{j})=\sum_{p=1}^{x+y+z}\vert M[i][p]-M[j][p]\vert  $$

4 $$ Cosine_{struct}(G_{i},G_{j})=\left(1-\frac{M[i]\cdot M[j]}{\vert M[i]\vert \vert M[j]\vert}\right)  $$

where,

- *M*[*i*] is complete annotation vector of gene *G*_*i*_.
- *M*[*i*][*p*] is the entry of the matrix for gene *G*_*i*_ corresponding to *p* ^*th*^ GO term where,
  if 1≤*p*≤*x*, then *p* ^*th*^ GO term is from Biological process ontology,
  if (*x*+1)≤*p*≤(*x*+*y*), then *p* ^*th*^ GO term is from Molecular function ontology,
  if (*x*+*y*+1)≤*p*≤(*x*+*y*+*z*), then *p* ^*th*^ GO term is from Cellular component ontology.
- |*M*[*i*]| = $\sqrt {\sum _{p=1}^{x+y+z}(M[i][p])^{2}}$.
- *M*[*i*]·*M*[*j*] is dot product of two annotation vector M[i] and M[j] corresponding to gene *G*_*i*_ and *G*_*j*_.

The adaptation of these three distance measures (Euclidean, city block and cosine distance) is motivated by the fact that these are some popular distances widely used as underlying similarity measures of different clustering algorithms as revealed by the literature survey [25, 26].

**Fig. 2:**
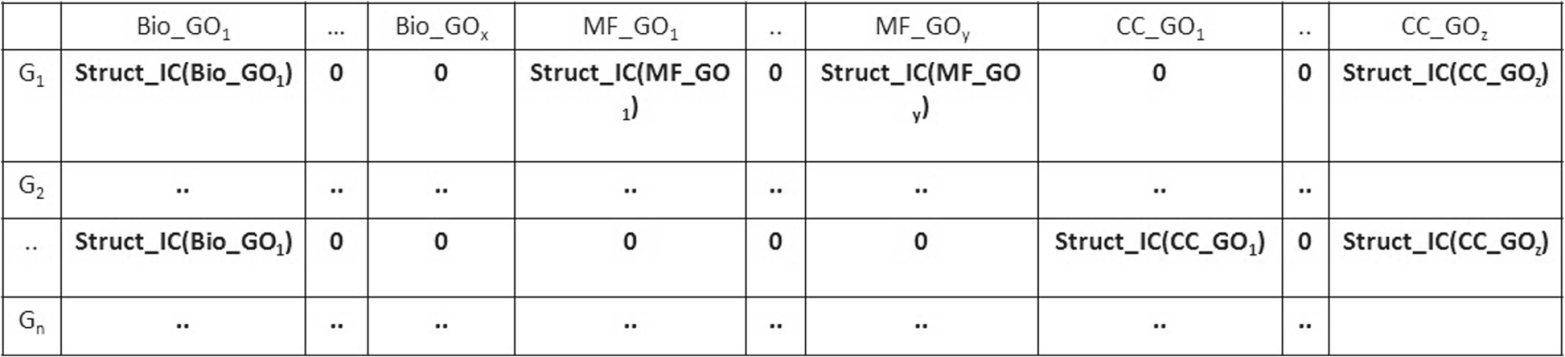
*Struct*_*IC*_ based gene-GO term annotation matrix representation

A sample *Struct*_*IC*_ based gene-GO term annotation matrix is shown in Fig. 2.

The formed *Struct*_*IC*_ based gene-GO term annotation matrix and the corresponding distance measures are used in gene selection process as described in next section.

#### Performing PAM clustering on gene-GO term data matrix and selecting most informative reduced gene space

Grouping of genes based on GO annotation data helps to capture different aspects of gene association patterns in terms of associated BP, CC and MF terms. Therefore, instead of performing clustering on gene expression data we have performed clustering on generated gene-GO term annotation matrix to identify functionally similar groups of genes. The Partitioning Around Medoids(PAM) [27] algorithm is a clustering algorithm related to the K-means algorithm and the medoid shift algorithm. K-means attempts to minimize the total squared error, while PAM minimizes the sum of dissimilarities between points which are in a single cluster with respect to the medoid, a point designated as the center of that cluster. In contrast to the K-means algorithm, PAM chooses any real data point from the existing cluster as the center. It is more robust to noise and outliers as compared to K-means because it minimizes a sum of general pairwise dissimilarities instead of a sum of squared Euclidean distances. Additionally it is very fast as K-means. Because of these reasons we have chosen PAM to perform clustering on gene-GO term annotation matrix utilizing three distances (euclidean, city block, cosine) alternatively to get functionally similar groups of genes. The steps of PAM clustering algorithm to get reduced gene space is given below,

1. Initializing ‘K’: According to “Input parameters for PAM” section select ‘p’ different values of ‘K’. So that, ∀*K*_*i*_,*i*∈ [1…*p*]. For each *K*_*i*_ perform **Step 2 to 7**.
2. Initializing solution: Randomly select *K*_*i*_ medoids(genes) from total available ‘n’ gene points.
3. Each non-medoid data point is assigned to it’s closest medoid. **(‘closest’ here is defined using any one of the distance measures as described in Eqs.** **2** **,** **3** **and** **4** **)**
4. For each medoid *m* and non-medoid data point *o*:
  Swap *m* and *o* and compute the cost(sum of distances of points to their medoid.).
5. Select the configuration with the lowest cost.
6. **Repeat Steps 3 to 5** until there is no change in the medoid.
7. Calculate Silhouette index value of finally obtained solution. Let us denote the Silhouette value as *Sil*(*Sol*_*i*_), where *Sol*_*i*_ is the finally obtained clustering solution by PAM having *K*_*i*_ medoids.
8. Choose *Sol*_*i*_ having *max*(*Sil*(*Sol*_*i*_)).
9. Validate the solution *Sol*_*i*_ with biological significance test.
10. Extract *K*_*i*_ number of medoids(representative genes) from *Sol*_*i*_. Suppose the size of set containing *K*_*i*_ medoids is represented by *n*_*m*_. It is the extracted reduced feature set.
11. Validate *n*_*m*_ features with biological significance test.

### Module 2: sample clustering over reduced feature(gene) space

After extracting the biologically significant and informative set of genes from module 1, in the next module the utility of obtained feature set is investigated through sample clustering. Suppose the dimension of original gene expression data is *d*×*n*, where *d* is the number of available samples and *n* is the number of available genes. After applying our proposed gene selection algorithm, the number genes in the reduced feature set is *n*_*m*_. So, the dimension of gene-expression data in the reduced space becomes *d*×*n*_*m*_. Existing literature [28, 29] proved the utility of multi-objective optimization(MOO) over single objective optimization in solving different real-life optimization problems. Inspired by this, in recent years several multi-objective optimization based clustering techniques are also developed in the literature [29, 30]. These approaches perform better than their single objective counter parts. Motivated by this, in the current study we have executed a multi-objective based clustering technique on samples of both original i.e. *d*×*n* and *d*×*n*_*m*_ gene expression matrices. Here sample classification problem is solved by clustering algorithm. A popular multi-objective optimization strategy, AMOSA(archived multi-objective simulated annealing) [28], is utilized as the backbone of the used multi-objective clustering technique. Here the main aim of clustering is to determine the homogeneous groups of samples by simultaneously optimizing a set of cluster validity indices capturing different cluster qualities. It has been shown in the literature that AMOSA excels in the field of MOO as compared to several other existing multi-objective evolutionary algorithms. The steps of AMOSA based proposed clustering technique are mentioned below,

#### String representation and archive initialization

In AMOSA [28] it uses the concept of string to represent each solution. At the beginning of execution it initializes the archive with some random solutions. Each archive member represents one complete clustering solution. Archive member length can vary from each other. Suppose in our chosen gene expression data set there are *d* number of samples and for each sample, expression value of *n* number of genes are there. *n* and *d* are specific to a data set.

#### Assignment of points and computation of objective functions

Once the archive members are initialized with some randomly selected cluster centroids from the set of input data points (here *d* samples represent *d* number of data points), assignment of rest of the *d* samples to different clusters is performed. This assignment can be done based on any standard distance measure. In this article we have used Euclidean distance for this purpose. The sample is assigned to that cluster with respect to which its Euclidean distance is the minimum. Next, we compute three cluster quality measures, XB index [31], PBM index [31], FCM index [31] which are used as three objective functions for each solution or string. The XB and FCM index values should be minimized and PBM index value should be maximized to get the optimal solution. Thereafter using the search methodology of AMOSA, we simultaneously optimize these three objective functions.

#### Search operators

In AMOSA perturbation operations are applied on current solution to generate new solutions to explore the search space effortlessly. In this work we have applied three different perturbation operations which are given as follows, A clustering solution can be changed in the three different ways,

1. Encoded cluster centers can be modified by some small values. By using Laplacian distribution we have randomly selected some values near the old values of cluster centers and then updated the existing centers.
2. Number of encoded clusters in a solution can be decreased by one. This is done by deleting a randomly selected cluster center from the given solution.
3. Number of encoded clusters in a solution can be increased by one. This is done by randomly selecting a point from the data set as the new cluster center and then inserting this in the solution.

Any one of these above mentioned search operators is applied on a string at a particular time.

#### Selecting best clustering solution from the Pareto Optimal front

It is the property of any MOO technique [28] to generate more than one non-dominating clustering solutions on it’s Pareto front. Each of these non-dominated solutions corresponds to a complete assignment of all data-points of chosen data set to different clusters. In the absence of additional information, any of those solutions can be selected as the optimal solution. In this approach we have selected the best solution using one internal cluster validity index, Silhouette index [31]. The solution having highest Silhouette index value is selected as the best solution.

### Chosen data sets and their description

We have applied our proposed unsupervised feature selection algorithm on gene-GO term annotation matrices and finally executed AMOSA based clustering on samples of gene expression data sets for 1) *Yeast* ^3^, 2) *Multiple tissues* ^4^ data sets. *Yeast* microarray data is a collection of 2884 genes (features) under 17 samples (time points). These 17 time points are categorized into two broad phases. Each of these two phases has four sub-phases named as G1, S, G2, and M [32]. Similarly, *Multiple tissues* data set comprises of 103 samples with 5565 genes(features). The samples are categorized into four normal tissue types of humans which are breast, prostate, lung and colon. In [32, 33] true class label information of *Yeast* data set is provided and described in detail. The true class label information for *Multiple tissues* is available in link^5^.

### Gene-GO term annotation matrix generation

We have used Gene Ontology Consortium^6^ to obtain the significant GO terms corresponding to mapped gene sets for both data sets. The chosen genomes for *Yeast* and *Multiple tissues* data sets are *Saccharomyces* *cerevisiae* and *Homosapiens*, respectively. Also the full GO tree^7^ was downloaded in.obo format. Originally in *Yeast* data set, 2260 number of genes out of 2884 genes are mapped to one or more GO terms under one or more gene ontologies (BP, MF, CC). For *Yeast* data set, the number of obtained significant GO terms is 166 (number of GO terms under BP is 100, under MF is 43, and under CC is 23). Similarly for *Multiple tissues* data set, 4673 number of genes out of 5565 genes are mapped to one or more GO terms. The obtained significant number of GO terms for *Multiple tissues* data set are 147 (number of GO terms under BP are 71, under MF are 42, and under CC are 34).

So the sizes of gene-GO term annotation matrices for *Yeast* and *Multiple tissues* data set are 2260×166 and 4673×147, respectively. Finally the entries of these matrices are calculated according to “Preparing gene-GO term annotation data for PAM based clustering” section.

## Results

### Setting of input parameters

#### Input parameters for PAM

For PAM clustering algorithm, priori information about the number of clusters (K) is needed. As the medoid of each cluster is selected as the member of reduced gene set, therefore the size of the reduced gene set is as same as the initial value of *K*. It is known that if no information about the number of clusters is given, then for *n* number of data points, the maximum number of clusters can be chosen as $\sqrt {n}$ [34]. According to that, for *Yeast* and *Multiple tissues* data sets, the maximum number of clusters can be $\sqrt {2260}$ or 48 and $\sqrt {4673}$ or 68, respectively. To explore different reduced gene sub-spaces, we have varied the value of *K* for both data sets as shown in Table 1.

**Table 1:** Chosen *K* values for PAM clustering algorithm

Data sets	K							
*Yeast*	5	10	20	30	40	50	-	-
*Multiple tissues*	5	10	20	30	40	50	60	70

#### Input parameters of AMOSA

We have executed AMOSA based clustering technique with the following parameter combinations:

$$\begin{array}{*{20}l} {}T_{min} =&\ 0.0001, T_{max} = 100, \alpha = 0.9, \text{HL} = 50, \text{SL} = 100\\ &\text{and}\ iter = 100. \end{array} $$

The parameter values are determined after conducting a thorough sensitivity study.

### Experiments conducted

1. At the beginning, we have applied three different well known and widely used distance measure (Euclidean, city block and cosine distance) based PAM algorithm on gene-GO term annotation data alternatively for both data sets. Among these three versions of PAM, one version is identified as best with respect to Silhouette index value of its corresponding produced clustering solution. The clustering solution of that version is used further to produce reduced gene space.
2. Once the reduced gene space is formed and biologically validated, then we have performed AMOSA [28] based clustering on samples of gene expression data over original and reduced gene spaces. After obtaining different clustering solutions we have compared their qualities based on three internal validity measures which are Silhouette index [35], Davies-Bouldin or DB index [36] and Dunn index [37].
3. Also we have performed a comparative study of our proposed feature selection based sample clustering approach with other existing approaches with respect to one external validity measure which is Classification Accuracy(%CoA).

### Objectives of experiments

1. To identify the most biologically informative feature(gene) set for clustering of samples in gene expression data.
2. To determine whether the generated reduced number of biologically significant genes leads to the improved performance for sample clustering.

### Chosen internal and external cluster validity measures for comparison

We have chosen three internal validity measures for comparison purpose. These are Silhouette index [35], DB index [36] and Dunn index [37]. For a good quality cluster the corresponding Silhouette and Dunn index values should be as large as possible where as smaller value of DB index signifies a better clustering solution. Also one external cluster quality measure, Classification Accuracy (%CoA), has been used to compare performance of proposed algorithm with other existing methods. As for both *Yeast* and *Multiple tissues* data sets, the true class label information are also available, therefore in order to verify our framework Classification Accuracy (%CoA) metric has been utilized.

## Discussion

### Discussion on results of *Yeast* data

After applying PAM based clustering algorithm on gene-GO term annotation matrix of *Yeast* data set utilizing three distances (Euclidean, city block and cosine) alternatively with different values of *K* as shown in Table 1, we have calculated the Silhouette index [35] values for different obtained clustering solutions corresponding to different *K* values. Those are reported in Table 2. It can be seen that PAM with Euclidean distance obtains optimal clustering solution with respect to Silhouette index for *K*=10. Similarly obtained optimal *K* values corresponding to city block and cosine distance based PAM are also highlighted in Table 2.

**Table 2:** Silhouette index values for clustering solutions produced by PAM with different values of *K*

Data set	K	Silho Eucli-PAM	Silho City-PAM	Silho Cosine-PAM
Yeast	5	0.3792	0.367	0.381
	**10**	**0.4531**	**0.452**	**0.442**
	20	0.4415	0.437	0.435
	30	0.4075	0.411	0.426
	40	0.40	0.421	0.423
	50	0.397	0.432	0.419
Multiple tissues	5	0.354	0.361	0.359
	10	0.383	0.372	0.368
	20	0.394	0.379	0.382
	30	0.406	0.394	0.392
	**40**	**0.4299**	**0.419**	0.404
	**50**	0.429	0.402	**0.418**
	60	0.415	0.398	0.416
	70	0.414	0.391	0.409

The data in boldface represents optimal value of ‘K’ i.e. dimension of gene space corresponding to optimal Silhouette index for all of three distance based PAM versions

If we closely observe the reported results in Table 2, we can see that for *Yeast* data set though the optimal value of *K* with respect to Silhouette index is same for all of the distances but the maximum value of this index is obtained by Euclidean based PAM. Therefore we consider the clustering solution obtained by Euclidean based PAM for further analysis.

To verify whether the clusters of the solution obtained by PAM (with euclidean distance) are biologically enriched or not, we have performed biological significance test with the help of GOTERMMAPPER^8^. The results for first two clusters out of three clusters for euclidean distance based PAM are shown in Table 3. In each table we have summarized significant GO terms shared by genes of corresponding cluster.

**Table 3:** Results for biological significance test: first two obtained clusters by PAM on *Yeast* data

*Cluster*	*GO term*	*Cluster %*	*Genome %*
Cluster 1	GO:0022625	57.1%	34.5%
245 genes	cytosolic large ribosomal subunit		
	GO:0042221	40.63%	28.29%
	response to chemical		
	GO:0006325	38.62%	22.86%
	chromatin organization		
	GO:0055085	47.94%	18.33%
	transmembrane transport		
Cluster 2	GO:0015934	44.1%	22.82%
156 genes	large ribosomal subunit		
	GO:0006974	37.74%	14.92%
	cellular response to DNA damage stimulus		
	GO:0006366	36.94%	18.58%
	transcription from RNA polymerase II promoter		
	GO:0006811	38.37%	19.47%
	ion transport		

**Fig. 3:**
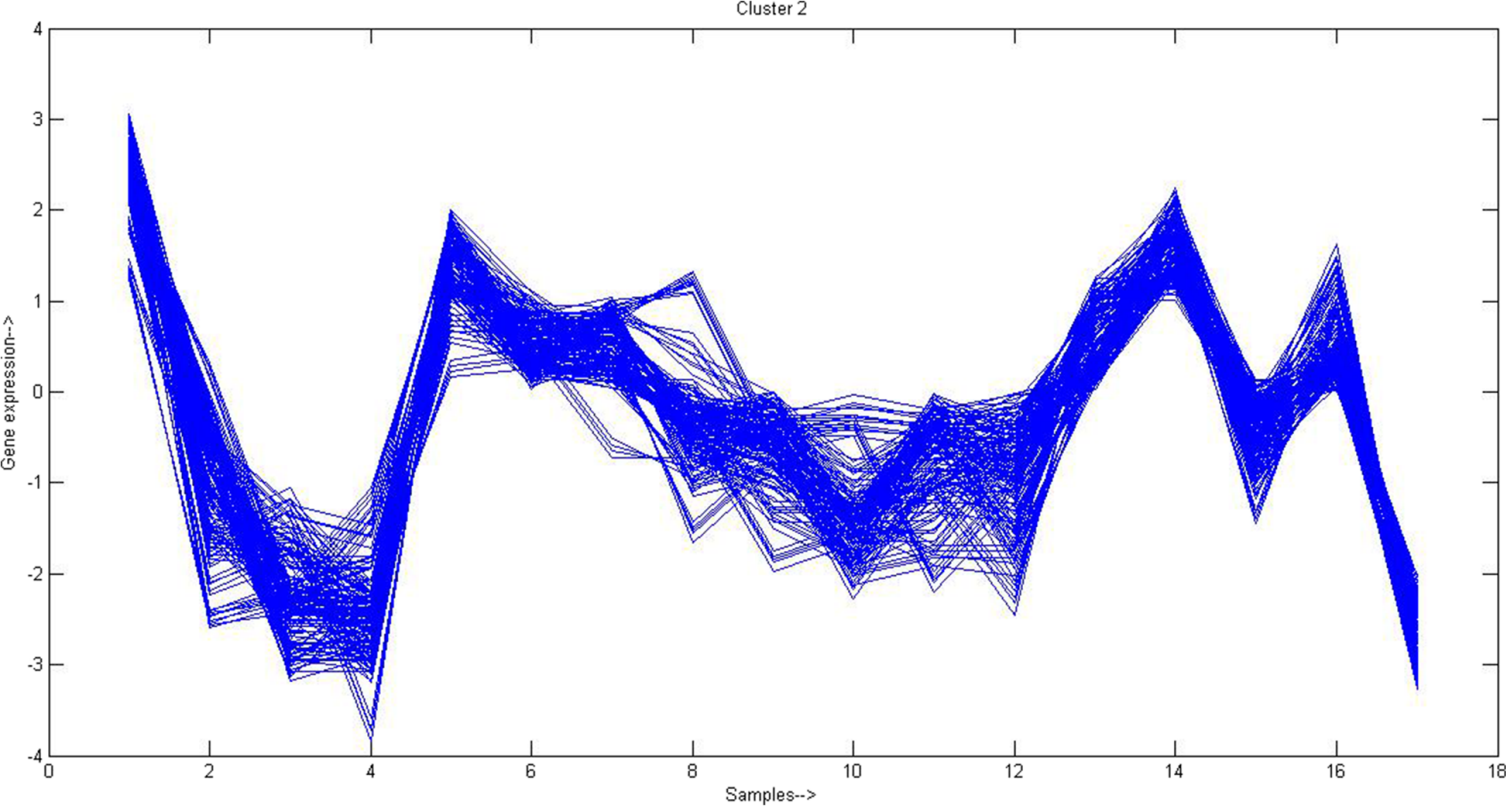
Cluster profile plot of one cluster (having 156 genes and 17 samples) after performing PAM based clustering on gene-GO term annotation matrix of *Yeast* dataset

For each GO term, the percentage of genes sharing that term among the genes of that cluster and among the whole genome have been reported. Results clearly signify that genes of same cluster share the higher percentage of GO terms compared to the whole genome. This indicates that the genes of a particular cluster are more involved in similar biological processes compared to the remaining genes of the genome. For rest 8 clusters the same behaviour was observed. Also to show the coherence between genes within same cluster the cluster profile plot is shown in Fig. 3 for one obtained cluster having 156 genes. In this plot the normalized expression values of genes within a cluster over all samples are plotted. The given cluster profile plot shows that genes within that cluster have good coherence among them for *Yeast* dataset. For other obtained clusters similar profile plots can be drawn to visualize the coherence among genes.

After biologically validating the solution obtained by euclidean based PAM algorithm, the most representative genes or medoids of different clusters are selected as genes of reduced gene set. The IDs of these 10 selected genes (as here *K*=10) are YLR068W, YMR143W, YDR379W, YPL150W, YGR152C, YFL008W, YBL084C, YDR361C, YLR325C, YDR165W. We have also evaluated the biological significance of these medoids(genes) using GOTERMMAPPER. We found all of them were annotated by one or more GO terms.

Once the reduced feature set is obtained, we perform AMOSA [28] based sample clustering over both original and reduced gene space. The obtained solutions are compared with each other with respect to some external cluster validity indices, namely Silhouette index [35], DB index [36] and Dunn index [37]. These results are shown in Table 5. Also, the results are plotted in graph as shown in Fig. 5. From both the table and figure it is clear that according to Silhouette, DB and Dunn indices, clustering of samples over reduced gene space is better than those over the full set. The clustering of samples over the reduced gene space contains more homogeneous clusters/partitions than the original space. The clusters obtained over the reduced gene space are more compact in shape and well-separated from each other.

Also we have performed comparative study with outcomes from other existing approaches on the same data sets with respect to one external validity measure, i.e., classification accuracy (%CoA). The results are shown in Table 6 and graphically shown in Fig. 6. In [20] %Coa of different classifiers after performing CLARANS based feature selection method were reported. They have also used these datasets with the corresponding true class label information for classification purpose. We have compared our proposed feature selection based sample clustering technique with reported approaches in [20] with respect to %CoA values. According to reported results in Table 6 and Fig. 6, it can be seen that our proposed method of sample clustering with reduced gene space provides best %CoA compared to other reported existing approaches. Also in our approach the dimension of reduced gene space is less than the reported reduced dimension of gene space in [20].

### Discussion on results of *Multiple tissues* data

Similar experiments are conducted for *Multiple tissues data*. The corresponding Silhouette index values of different clustering solutions after performing three distance based PAM on genes of gene-GO term annotation matrix for this data set with different chosen *K* values are shown in Table 2. From this table, we can see that the best clustering solution is obtained for *K*=40 by Euclidean based PAM. The optimal *K* values are also highlighted for other distance based PAM. But among all these three distances, Euclidean based PAM produces optimal solution having maximum Silhouette index value. Therefore, similar to *Yeast* data set, we have considered optimal clustering solution obtained by Euclidean based PAM for *Multiple tissues* data set for further analysis.

**Fig. 4:**
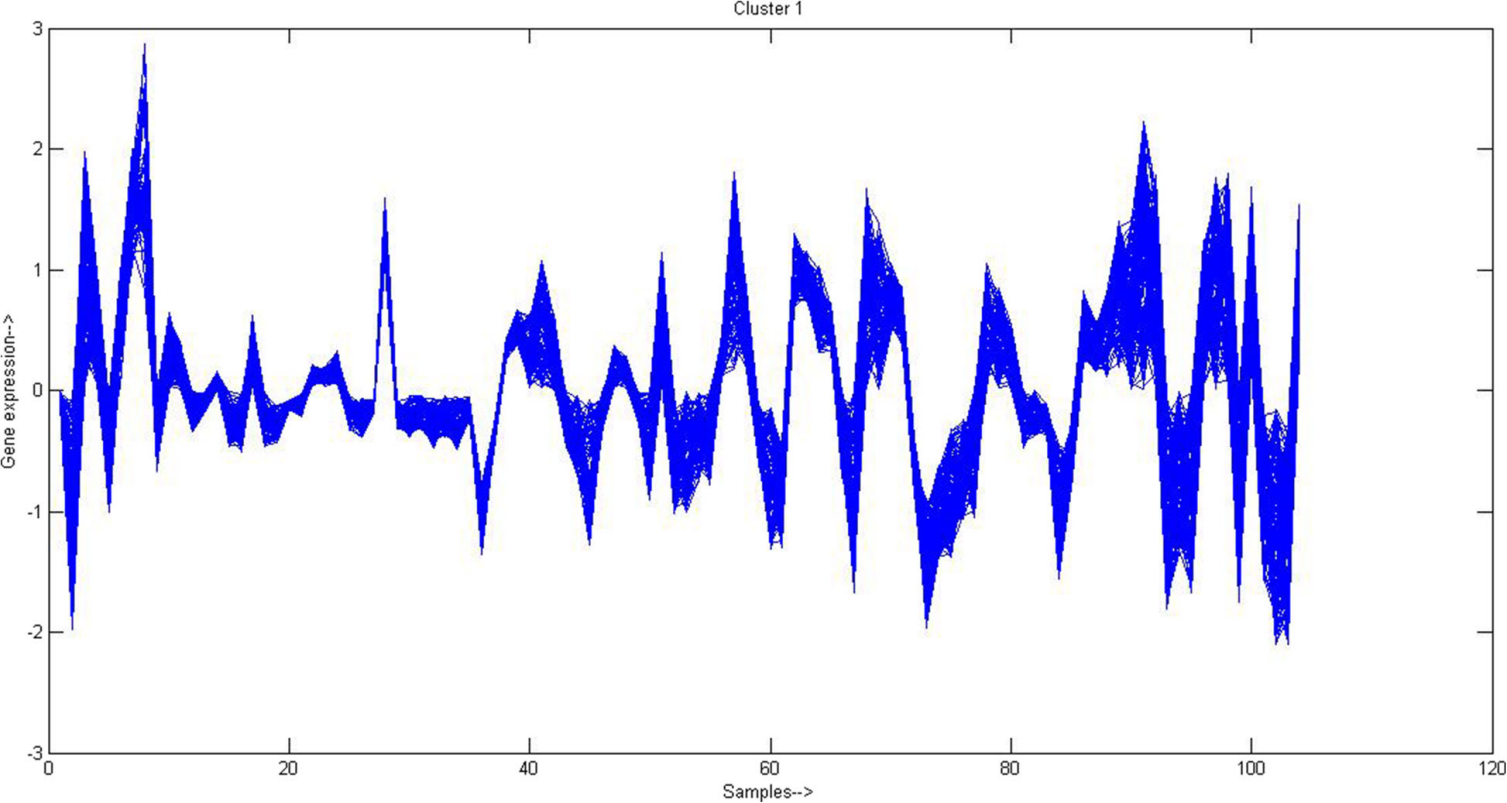
Cluster profile plot of one cluster (having 102 genes and 103 samples) after performing PAM based clustering on gene-GO term annotation matrix of *Multiple tissue* dataset

Similar to *Yeast* data set we have cross validated the obtained clusters of solution with *K*=40 by euclidean based PAM using biological significance test with the help of GOTERMMAPPER^9^. For first two clusters the biological significance test outcomes are shown in Table 4. Similar test was done for other 38 clusters. Also in Fig. 4, cluster profile plot for one obtained cluster having 102 number of genes is shown. From the plot it is clearly evident that genes within that cluster have good coherence among them. For other obtained clusters, the coherence can be checked similarly with the help of cluster profile plot. Next we form the reduced feature set by considering only the medoid genes from each of 40 clusters. The IDs of these selected medoids/genes are CCL22, CD8B1, CORO2B, CSTF1, EPHX1, GA17, KIAA0350, KIAA0460, KIAA0980, RAB9P40, RPL10A, SEC22L1, SMARCC1, STAC, TAF1C, HIPK3, TMEM1, TNFRSF25, ZFR, TPM3, HIST2H2AA, HOXC5, ISGF3G, MYLK, ORM1, PSMD12, PTGER1, RECK, RGS3, SEC31L1, ZNF629, NPIP, KIAA0792, BAT2D1, DC12, WBSCR20C, ST5, MAPK1, ALM2-AKAP2, SEPW1. During biological significance test of this feature set using GOTERMMAPPER, we found that all of them are annotated using one or more GO terms.

**Table 4:** Results for biological significance test: first two obtained clusters by PAM on *Multiple tissues* data

*Cluster*	*GO term*	*Cluster %*	*Genome %*
Cluster 1	GO:0009987	73.00%	59.72%
102 genes	cellular process		
	GO:0008152	75.00%	46.46%
	metabolic process		
	GO:0050789	69.00%	36.75%
	regulation of biological process		
	GO:0050896	67.00%	26.47%
	response to stimulus		
	GO:0032501	55.00%	16.69%
	multicellular organismal process		
Cluster 2	GO:0043170	52.48%	35.46%
107 genes	macromolecule metabolic process		
	GO:0009058	44.55%	22.22%
	biosynthetic process		
	GO:0032501	40.59%	16.69%
	multicellular organismal process		
	GO:0007154	32.67%	19.46%
	cell communication		
	GO:0007275	28.71%	11.47%
	multicellular organismal development		

**Fig. 5:**
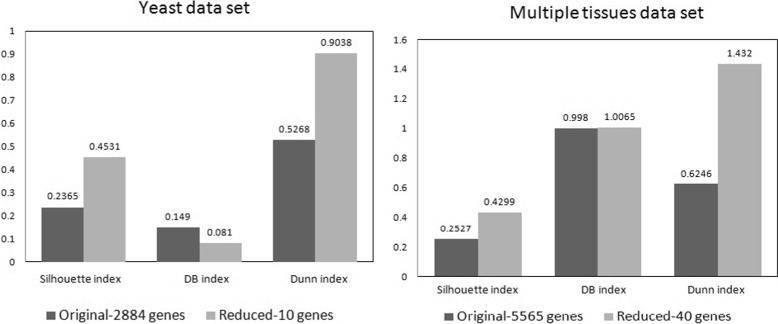
Graphical comparative analysis of AMOSA based sample clustering outcomes with respect to three internal cluster validity indices

After obtaining the reduced gene space, AMOSA based sample clustering is performed on *Multiple tissues* gene expression data set over both original and reduced gene space. The comparative analysis is shown in Table 5. Also, these results are graphically shown in Fig. 5. According to this table and figure, from the obtained results, it is clearly evident that the reduced set of genes for this data set provides better clustering solution with respect to Silhouette and Dunn index values in almost all cases. With respect to DB index value, the quality of clustering of samples over original gene space is slightly better than that of the reduced gene space. But in this case the difference is very negligible (by the value 0.0085). As the dimension of gene space reduces by a large scale, it significantly reduces the computational costs of the sample clustering/classification process.

**Table 5:** Comparative analysis of AMOSA based sample clustering outcomes with respect to three internal validity indices

Data set	Genes(features)	Samples	Silho	DB	Dunn
Yeast	2884(Original)	17	0.2365	0.149	0.5268
	10(Reduced)		**0.4531**	**0.081**	**0.9038**
Multiple tissues	5565(original)	103	0.2527	**0.998**	0.6246
	40(Reduced)		**0.4299**	1.0065	**1.432**

The obtained optimal values for Silhouette, DB and Dunn index for both datasets are represented in bold font

**Fig. 6:**
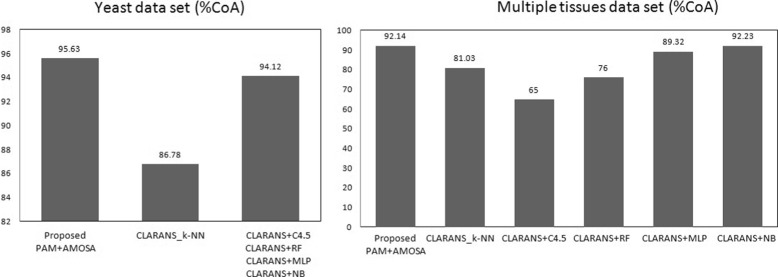
Graphical comparative analysis of our proposed feature selection based sample clustering technique with other existing techniques

Again the results are compared with the reported results of [20] with respect to %CoA values. These results are reported in Table 6 and graphically shown in Fig. 6. From the obtained results we have seen that our approach provides better %CoA values than all other approaches except *CLARANS*+*NB* approach. That approach outperforms our approach by a small scale (0.09%). Here also our obtained gene space dimension is lower compared to the obtained dimension in [20].

**Table 6:** The comparative results of our proposed feature selection based sample clustering technique with other existing techniques

Data set	Number of genes	Algorithms	%CoA
Yeast	10	Proposed(PAM+AMOSA)	**95.63**
	15	CLARANS+k-NN	86.78
		CLARANS+C4.5	94.12
		CLARANS+RF	94.12
		CLARANS+MLP	94.12
		CLARANS+NB	94.12
Multiple tissues	40	Proposed(PAM+AMOSA)	92.14
	42	CLARANS+k-NN	81.03
		CLARANS+C4.5	65.0
		CLARANS+RF	76.0
		CLARANS+MLP	89.32
		CLARANS+NB	**92.23**

The obtained optimal (maximum) Classification accuracy (%CoA) for both datasets are represented in bold font

So, overall we can say that our proposed method provides a most informative and discriminative reduced set of genes(features) compared to existing approaches for both data sets and this argument is supported by most of the cases in our conducted comparative analysis.

## Conclusions

In this paper we have proposed an unsupervised feature selection technique utilizing available biological knowledge extracted from GO. Here as biological knowledge we have utilized gene annotation data, where each gene is represented as structural IC based gene-GO term annotation vector which intuitively forms gene-GO term annotation matrix for a selected data set. The proposed method of performing PAM based clustering on annotation matrix to identify biologically informative and discriminative set of features(genes) is a contribution of the current work. To visualize the coherence between genes of obtained clusters, cluster profile plot is used for both datasets. Also we have validated the selected features with proper biological significance test.

Finally AMOSA based clustering is performed on samples on gene-expression data with reduced gene set. From the obtained results we have observed that utilizing biological knowledge in feature selection step not only reduces the dimension of the gene space in large scale but also improves the classification accuracy of samples.

In future we would like to apply some other clustering algorithms in place of PAM to identify the appropriate gene subset where the dimensionality of gene subset can be determined automatically. We are currently working in that direction.

## Endnotes

^1^ http://www.geneontology.org/

^2^ http://www.geneontology.org/page/download-ontology

^3^ http://arep.med.harvard.edu/

^4^ http://portals.broadinstitute.org/cgi-bin/cancer/datasets.cgi

^5^ http://portals.broadinstitute.org/cgi-bin/cancer/datasets.cgi

^6^ http://www.geneontology.org/

^7^ http://www.geneontology.org/page/download-ontology

^8^ http://go.princeton.edu/cgi-bin/GOTermMapper

^9^ http://http://go.princeton.edu/cgi-bin/GOTermMapper

## Abbreviations

AMOSA: Archived multi objective simulated annealing
BP: Biological process
CC: Cellular component
CLARANS: Clustering large applications based upon RANdomized search
CoA: Classification accuracy
ERGS: Effective range based gene selection
GS: Gene selection
GO: Gene ontology
IC: Information content
MF: Molecular function
MOO: Multi objective optimization
MRMR: Minimum redundancy maximum relevance
NB: Naive bayes
PAM: Partitioning around medoids
RFA: Recursive Feature Addition
SemiFeaClustMOO: Multi objective semisupervised clustering as well as feature-selection technique
*Struct*_*IC*_: Structure based information content

## References

[1] de Souto MC, Costa IG, de Araujo DS, Ludermir TB, Schliep A (2008). Clustering cancer gene expression data: a comparative study. BMC Bioinformatics.

[2] Mukhopadhyay A, Maulik U, Bandyopadhyay S (2010). On biclustering of gene expression data. Curr Bioinforma.

[3] Xing EP, Jordan MI, Karp RM (2001). Feature selection for high-dimensional genomic microarray data. proc. of the Eighteenth International Conference on Machine Learning (ICML 2001), Vol. 1.

[4] Xiong M, Fang X, Zhao J (2001). Biomarker identification by feature wrappers. Genome Res.

[5] Blum AL, Langley P (1997). Selection of relevant features and examples in machine learning. Artif Intell.

[6] Dy JG, Brodley CE, Kak A, Broderick LS, Aisen AM (2003). Unsupervised feature selection applied to content-based retrieval of lung images. IEEE Trans Pattern Anal Mach Intell.

[7] Chagoyen M, Carmona-Saez P, Gil C, Carazo JM, Pascual-Montano A (2006). A literature-based similarity metric for biological processes. BMC Bioinformatics.

[8] Del Pozo A, Pazos F, Valencia A (2008). Defining functional distances over gene ontology. BMC Bioinformatics.

[9] Lim WK, Wang K, Lefebvre C, Califano A (2007). Comparative analysis of microarray normalization procedures: effects on reverse engineering gene networks. Bioinformatics.

[10] Fröhlich H, Speer N, Poustka A, Beißbarth T (2007). Gosim–an r-package for computation of information theoretic go similarities between terms and gene products. BMC Bioinformatics.

[11] Wolting C, McGlade CJ, Tritchler D (2006). Cluster analysis of protein array results via similarity of gene ontology annotation. BMC Bioinformatics.

[12] Martin D, Brun C, Remy E, Mouren P, Thieffry D, Jacq B (2004). Gotoolbox: functional analysis of gene datasets based on gene ontology. Genome Biol.

[13] Yang K, Cai Z, Li J, Lin G (2006). A stable gene selection in microarray data analysis. BMC Bioinformatics.

[14] Tsai YS, Lin CT, Tseng GC, Chung IF, Pal NR (2008). Discovery of dominant and dormant genes from expression data using a novel generalization of snr for multi-class problems. BMC Bioinformatics.

[15] Liu Q, Sung AH, Chen Z, Liu J, Huang X, Deng Y (2009). Feature selection and classification of maqc-ii breast cancer and multiple myeloma microarray gene expression data. PloS ONE.

[16] Chandra B, Gupta M (2011). An efficient statistical feature selection approach for classification of gene expression data. J Biomed Inform.

[17] Gunavathi C, Premalatha K (2014). Performance analysis of genetic algorithm with knn and svm for feature selection in tumor classification. Int J Comput Electr Autom Control Inf Eng.

[18] Saha S, Alok AK, Ekbal A (2016). Use of semisupervised clustering and feature-selection techniques for identification of co-expressed genes. IEEE J Biomed Health Inform.

[19] Qi J, Tang J (2006). Gene ontology driven feature selection from microarray gene expression data. proc. of the 2006 IEEE Symposium on Computational Intelligence in Bioinformatics and Computational Biology, CIBCB 2006.

[20] Mitra S, Ghosh S (2012). Feature selection and clustering of gene expression profiles using biological knowledge. IEEE Trans Syst Man Cybern Part C (Appl Rev).

[21] Ghosh S, Mitra S (2012). Gene selection using biological knowledge and fuzzy clustering. proc. of IEEE International Conference on Fuzzy Systems.

[22] Resnik P. Using information content to evaluate semantic similarity in a taxonomy.arXiv preprint cmp-lg/9511007. 1995.

[23] Teng Z, Guo M, Liu X, Dai Q, Wang C, Xuan P (2013). Measuring gene functional similarity based on group-wise comparison of go terms. Bioinformatics.

[24] Guzzi PH, Mina M, Guerra C, Cannataro M (2012). Semantic similarity analysis of protein data: assessment with biological features and issues. Brief Bioinform.

[25] Wang H, Wang W, Yang J, Yu PS (2002). Clustering by pattern similarity in large data sets. proc. of the 2002 ACM SIGMOD International Conference on Management of Data.

[26] Paul S, Maji P (2014). City block distance and rough-fuzzy clustering for identification of co-expressed micrornas. Mol BioSyst.

[27] Kaufman L, Rousseeuw PJ (1990). Partitioning around medoids (program pam). Finding groups in data: an introduction to cluster analysis.

[28] Bandyopadhyay S, Saha S, Maulik U, Deb K (2008). A simulated annealing-based multiobjective optimization algorithm: Amosa. IEEE Trans Evol Comput.

[29] Alok AK, Saha S, Ekbal A (2016). Multi-objective semi-supervised clustering for automatic pixel classification from remote sensing imagery. Soft Comput.

[30] Li L, Jiao L, Zhao J, Shang R, Gong M (2017). Quantum-behaved discrete multi-objective particle swarm optimization for complex network clustering. Pattern Recog.

[31] Bandyopadhyay S, Saha S. Unsupervised Classification: Similarity Measures, Classical and Metaheuristic Approaches, and Applications: Springer Science & Business Media; 2012.

[32] Cho SB, Yoo SH (2006). Fuzzy bayesian validation for cluster analysis of yeast cell-cycle data. Pattern Recognit.

[33] Cho RJ, Campbell MJ, Winzeler EA, Steinmetz L, Conway A, Wodicka L, Wolfsberg TG, Gabrielian AE, Landsman D, Lockhart DJ (1998). A genome-wide transcriptional analysis of the mitotic cell cycle. Molecular Cell.

[34] Bezdek JC, Pal NR (1998). Some new indexes of cluster validity. IEEE Trans Syst Man Cybern B (Cybern).

[35] Rousseeuw PJ (1987). Silhouettes: a graphical aid to the interpretation and validation of cluster analysis. J Comput Appl Math.

[36] Davies DL, Bouldin DW (1979). A cluster separation measure. IEEE Trans Pattern Anal Mach Intell.

[37] Dunn JC (1974). Well-separated clusters and optimal fuzzy partitions. J Cybern.

